# Toward a taxonomic model of attention in effortful listening

**DOI:** 10.3758/s13415-017-0513-0

**Published:** 2017-05-31

**Authors:** Daniel J. Strauss, Alexander L. Francis

**Affiliations:** 1 Systems Neuroscience and Neurotechnology Unit, Neurocenter, Faculty of Medicine, Saarland University & School of Engineering, Building 90.5, 66421 htw saar, Homburg/Saar Germany; 2 Speech Perception and Cognitive Effort Laboratory Department of Speech, Language & Hearing Sciences, Purdue University, West Lafayette, IN USA; 3 Leibniz-Institute for New Materials, Saarbruecken, Germany; 4 Key Numerics GmbH – Neurocognitive Technologies, Saarbruecken, Germany

**Keywords:** Listening effort, Attention, Modeling, Speech perception

## Abstract

In recent years, there has been increasing interest in studying listening effort. Research on listening effort intersects with the development of active theories of speech perception and contributes to the broader endeavor of understanding speech perception within the context of neuroscientific theories of perception, attention, and effort. Due to the multidisciplinary nature of the problem, researchers vary widely in their precise conceptualization of the catch-all term *listening effort*. Very recent consensus work stresses the relationship between listening effort and the allocation of cognitive resources, providing a conceptual link to current cognitive neuropsychological theories associating effort with the allocation of selective attention. By linking listening effort to attentional effort, we enable the application of a taxonomy of external and internal attention to the characterization of effortful listening. More specifically, we use a vectorial model to decompose the demand causing listening effort into its mutually orthogonal external and internal components and map the relationship between demanded and exerted effort by means of a resource-limiting term that can represent the influence of motivation as well as vigilance and arousal. Due to its quantitative nature and easy graphical interpretation, this model can be applied to a broad range of problems dealing with listening effort. As such, we conclude that the model provides a good starting point for further research on effortful listening within a more differentiated neuropsychological framework.

## Introduction

Under optimal circumstances, understanding speech can seem automatic and effortless. However, conditions are rarely optimal, and a wide variety of factors have been identified that, individually or in combination, may serve to make understanding speech more difficult. Characterizations of listening difficulty vary, centering around measures of intelligibility (i.e., number of keywords recognized correctly) but also secondarily encompassing measures of effort and satisfaction or acceptability. Although the source of difficulty may be associated with the talker, the listener, or the communication channel (or some combination thereof) (Mattys, Davis, Bradlow, & Scott, 2012), until recently, the primary focus has been on intelligibility, under the general assumption that if intelligibility can be improved, listening will become less effortful and more acceptable. Recently, however, researchers building on earlier work showing effects of noise on recall independent of intelligibility, e.g., Schneider, Daneman, and Pichora-Fuller (1995) have become increasingly concerned with cases in which listening effort may change even while intelligibility remains constant (e.g., Sarampalis, Kalluri, Edwards, & Hafter, 2009), and in cases in which measures of listening effort may be *more* informative than measures of performance (Desjardins and Doherty 2014). Such cases suggest that effort is at least partially independent of intelligibility, and that it may be perhaps equally significant for investigating speech perception.

Although there is a long tradition of research in cognitive effort more broadly, with a few exceptions (e.g., Nusbaum & Schwab, 1997; Surprenant, 1999) speech perception researchers have only recently begun to consider the theoretical implications of the observed need to commit effort to understand speech, e.g., Downs (1982), Pichora-Fuller and Singh (2006), Pichora-Fuller et al. (2016). This increased emphasis on explaining the effort demanded by listening to speech accords well with the increasing integration of theories of speech perception with broader cognitive neuroscientific theories of perception, attention, and effort. However, current conceptualizations of listening effort do not yet necessarily reflect the complexity of the current state of cognitive neuroscience research in these areas. To address this lack, in this article we propose a conceptual model of the contribution of varieties of attention to the determination of listening effort more broadly considered.

In the remainder of this section, we summarize the argument that the study of listening effort is significant in its own right, and make the case for considering it with particular reference to the cognitive mechanism of attention. In subsequent sections, we elaborate on two hypothetical examples of contexts in which listening is effortful. We highlight the need to distinguish between the external and internal dimensions of listening effort and link this distinction to similar distinctions in current, neurally motivated multi-dimensional models of selective attention. The third section consists of the mathematical description of the model itself, while the fourth section contains a discussion of some theoretical insights and motivations for further research that derive directly from the model. We conclude by relating the model to existing theories of attention and listening effort.

### Listening Effort Matters

The study of listening effort is significant for a variety of reasons. On the one hand, behavioral and psychophysiological measures frequently associated with listening effort such as pupil dilation, response time, dual-task interference, and various neuroimaging techniques may provide a more sensitive estimate of task demand than do simple measures of performance, and therefore may enable more sophisticated investigations of the neurocognitive mechanisms underlying listening and speech perception (cf. Strauss, Corona-Strauss, & Froehlich, 2008; Strauss et al., 2010; Bernarding, Strauss, Hannemann, Seidler, & Corona-Strauss, 2013; Wild et al., 2012; Wisniewski et al., 2015; Kramer, Teunissen, & Zekveld, 2016; Mackersie & Calderon-Moultrie, 2016). In particular, there are cases, especially associated with the effects of aging and hearing impairment, in which measures related to listening effort are more sensitive than simple performance measures such as proportion of words correctly recognized, and therefore such measures can be more relevant than other performance measures in cases in which the problem is no longer whether something can be understood, but rather how easy it is to do so (Desjardins & Doherty, 2014; Tun, McCoy, & Wingfield, 2009, Bernarding, Strauss, Hannemann, Seidler, & Corona-Strauss, 2017).

On the other hand, listening effort may in itself be an important factor in many aspects of human behavior, and thus may constitute an important object of study in and of itself (cf. Pichora-Fuller et al., 2016). For example, a person may choose to avoid going out with friends to a noisy restaurant in which communication is perceived to be too effortful even when it is nevertheless still eminently possible, and such decisions may have a significant effect on individuals’ quality of life (Pichora-Fuller, Mick, & Reed, 2015; Hornsby, Naylor, & Bess, 2016). Similarly, research on the use of hearing aids suggests that the effort or cognitive demand of listening to speech may be a deciding factor in some listeners’ preferences for particular signal processing strategies (Sarampalis, 2009; Souza, Arehart, & Neher, 2015; Bernarding et al., 2017).

Thus, investigations of listening effort will contribute to the development of better methods for studying speech perception, and will facilitate research into the challenges faced by people with hearing impairment and other difficulties that affect speech perception as well improving methods for seeking solutions to those challenges. However, none of these goals can be achieved without a better understanding of what listening effort is.

### Listening Effort as Attentional Effort

Researchers vary widely in terms of their precise conceptualization of listening effort (McGarrigle et al. 2014), but we will take as our starting point the consensus paper recently published as part of a special issue on listening effort by Pichora-Fuller et al. (2016) that defines mental effort as: “The deliberate allocation of mental resources to overcome obstacles in goal pursuit when carrying out a task.” (p. 11S). Of particular interest for our purposes is the emphasis on the allocation of cognitive resources. While much of the work in this area specifically focuses on *working memory* capaas representative of the limited mental resource that must be allocated deliberately (e.g., Souza et al., 2015, though cf. Füllgrabe & Rosen 2016), other cognitive mechanisms may also be relevant. Indeed, Pichora-Fuller and colleagues explicitly incorporate the work of Kahneman (e.g., Kahneman, 1973) who links *attention* with effort (see also Sarter, Gehring, & Kozak, 2006). While a thorough exposition of the relationship between working memory and attention is beyond the scope of the present article, it is clear that both attention and working memory are limited in capacity (Fougnie & Marois 2006), and although they may be distinguishable, they appear to interact to a very significant degree both behaviorally and neurally in the auditory domain (Backer, Binns, & Alain, 2015; Huang, Seidman, Rossi, & Ahveninen, 2013; Yurgil & Golob, 2013) and more generally (Kiyonaga & Egner, 2013; Nobre et al., 2004).

In particular, the availability of working memory capacity seems to modulate susceptibility to interference from auditory distractors (Fairnie, Moore, & Remington, 2016; Francis, 2010; Bertoli & Bodmer, 2013), a key function of attention that is particularly relevant in research on listening effort. We focus here on the relationship between listening effort and attention because of the close relationship between attention and the control of information processing. We do not distinguish between different *varieties* or *applications* of attention such as vigilance, selective and divided attention, or even such overarching terms as *executive control*. While such distinctions are clearly relevant for more in-depth investigations of attention demanding behavior, for our present purposes it is sufficient to characterize attention as the mechanism that serves to select stimuli for further processing, and, once selected, to modulate how well relevant information is processed (Strauss et al., 2010; Chun, Golomb, & Turk-Browne, 2011; Wild et al., 2012). Attentional effort, then, is the “cognitive incentive” (see Sarter et al., 2006) or “opportunity cost” (Kurzban, Duckworth, Kable, & Myers, 2013; Westbrook & Braver, 2015; Kurzban, 2016) of allocating attention to select stimuli and/or modulate their relevance in pursuit a particular goal, see also Eckert, Teubner-Rhodes, & Vaden, (2016) (please find a detailed discussion of “cognitive incentive” vs. “opportunity cost” interpretations in “Relation to other selected models and concepts”).

Following Westbrook and Braver (2015), we further distinguish between exogenous influences on attentional allocation, which are likely effortless (e.g., the capture of attention by a sudden loud sound) and endogenous direction of attention in a goal-directed manner. In terms of the field of auditory perception more specifically, we are building on the ternary distinction made by Shinn-Cunningham and colleagues (Shinn-Cunningham 2008; Shinn-Cunningham and Best 2008) between *object formation*, *object selection*, and *coping mechanisms* such as “filling in” and “recalling from memory” corresponding respectively to (1) exogenously controlled, externally directed, (2) endogenously controlled, exogenously directed, and (3) endogenously controlled, endogenously directed if only on the last two because evidence suggests that object formation may proceed more or less automatically, i.e., without the necessary application of endogenous control (Alain 2003).

We note also that even among explicitly endogenously controlled attentional processes there is a potential distinction to be made between consciously exerting effort (the sensation of “trying harder”) and the involuntary application of cognitive processes that, although they are not necessarily amenable to conscious awareness, demand effort when in operation (e.g., the increased demand introduced by the presence of competing lexical items in an response array, Kuchinsky et al., 2014). In general, it seems to be the second sort of mechanism that is of greatest relevance in current studies of listening effort, though the distinction is one that may warrant further consideration in future work.

### Distinct Domains of Attentional Allocation

The effort arising from the endogenous allocation of limited attentional capacity is central to discussions of listening effort associated with hearing impairment and speech perception in adverse, i.e., noisy or reverberant, conditions (Pichora-Fuller et al. 2016; Shinn-Cunningham and Best 2008) but is also invoked when considering perception of speech that is inherently difficult to process such as speech in a foreign language or spoken with a foreign accent (Lecumberri & Cooke, 2006; Lecumberri, Cooke, & Cutler, 2010; Hoen et al., 2007), or in an utterance that is syntactically complex (Carroll and Ruigendijk 2013; Wingfield et al. 2006).

Thus, listening tasks that are effortful seem to be distinguishable according to the source of the demand on processing resources: some sources of effort seem to require the distribution of attention to representations of phenomena that lie outside the listener, while other attention-demanding representations arise more centrally. As we will argue, the distinction between the effort associated with directing attention to external vs. internal information may be of particular importance (as in the difference between the effort involved in attending to one voice among many competitors vs. that of selecting one lexical item out of a field of many alternative possibilities, (cf. Kuchinsky et al., 2014).

Some version of this distinction has begun to appear quite explicitly in more recent discussions of listening effort, e.g., Edwards (2016) and Kuchinsky et al. (2014), but it is also evoked directly in research on auditory attention more generally, e.g., Shinn-Cunningham and Best (2008), Shinn-Cunningham (2008) and Francis (2010). For example, Kuchinsky et al. (2014) distinguish between task demands that increase with increasing masking and those that increase due to increased lexical response competition. Furthermore, they observe differences in patterns of pupil dilation (putatively reflecting effort) associated with these different sources of task demand although they do not explicitly attribute such differences in pupil dilation to differences in underlying mechanisms of processing. Similarly, Picou and Ricketts (2014) manipulated the depth of processing (cf. Craik & Lockhart, 1972) required by a secondary task while listeners performed a primary word recognition in noise task. While their specific findings do not bear directly on the potential distinction between externally and internally directed attention, the design of the experiment itself acknowledges such a possibility.

From a more theoretical perspective, in motivating the FUEL model (Pichora-Fuller et al. 2016) make a similar distinction when they refer separately to cases in which “listeners may expend more mental effort to direct attention to and concentrate on one or more sound sources of interest.” and cases in which “Individuals may also need to allocate more cognitive capacity to comprehend, remember, and respond to the auditory objects and events that they have perceived.” (p. 6S). Associated with the FUEL model, Edward’s graphic depiction of the augmented *Ease of Language Understanding Model (ELU)* explicitly represents two different loci of effort, one more central and one more peripheral (Edwards, 2016, Fig. 2). However, perhaps because the focus of these models is on the limited resources that are deployed or consumed (especially working memory capacity), and both external and internal allocation of attention very likely depend at least partly on the availability of working memory capacity (Kiyonaga and Egner 2013; Nobre et al. 2004), the implications of this distinction are not fully explored in these works. Nevertheless, a distinction between the distribution of processing capacity toward more perceptual vs. more central representations is pervasive in the field of hearing science, and in the literature on attentional processing more generally.

As suggested by Picou and Ricketts (2014) this distinction is reminiscent of the more general cognitive psychological concept of *levels of processing* (e.g., Craik & Lockhart, 1972) and similar sorts of distinctions between more perceptual and more central kinds of processes are well represented in recent characterizations of visual attention within the context of general theories of attention as well, especially, e.g., in the work of Lavie and colleagues (Lavie, 2000; Lavie, Hirst, De Fockert, & Viding, 2004), and Chun and colleagues (Chun et al., 2011). Finally, a systems neuroscience perspective also suggests to make this distinction explicit as different neural circuits are involved, e.g., see Engel, Fries, & Singer (2001), Raz and Buhle (2006), Fougnie (2008), Chun et al. (2011). Thus, there are many reasons for considering a distinction between internal and external attention and, as we show in the examples in the next section, such a distinction also corresponds well to subjective experiences with effortful listening.

## Two distinct dimensions of attention in effortful listening

Suppose you are in a poster session and you are in front of an interesting piece of work. The presenter is explaining to you what has been done. One could imagine the following very distinct situations that make listening effortful in this context: In listening situation (a) the area of the poster is very crowded, there is a lot of talking and perhaps some chuckling at varying intensities from the immediate audience and also from the neighboring posters. It is effortful for you to follow the explanations of the presenter because you must allocate attention to the speech of interest while filtering out all sorts of competing auditory information.

In contrast, in listening situation (b) you find yourself alone with the presenter in a more or less ideal auditory environment. It is quiet and the room is large enough to be comparatively non-reverberant. However, the presenter is using unfamiliar technical terminology and awkward sentence constructions. You can still make sense of the talk by extrapolating from the semantic context and the occasional familiar Greek or Latin root to puzzle out the meaning of the unknown words, and by mentally reconfiguring awkward or ambiguous sentences into something easier to parse. In this case, the effort arises from the need to allocate attention to internal representations of concepts, words and grammatical structures in order to achieve an accurate interpretation and resolve potential ambiguities or uncertainties (cf. Altmann & Steedman, 1988; Chambers, Tanenhaus, & Magnuson, 2004).

Although both situations involve the application of *listening effort* as it is currently defined (McGarrigle et al. 2014; Pichora-Fuller et al. 2016), there seems to be something qualitatively different about the types of attention required for each task, and the aforementioned behavioral and neuropsychological evidence supports this distinction as well. Such differences become especially relevant when considering the increasing interest in developing psychophysiological measurements for assessing listening effort objectively. To begin to address this issue, here we present a taxonomic model of attention in effortful listening.

Following Chun et al. (2011), we distinguish between externally directed (perceptual) attention and internally directed (central) attention. Here “external attention” refers to the active, endogenously directed selection and modulation of externally generated (sensory) objects (see “External dimension of listening effort”) whereas “internal attention” refers to the active, endogenously directed selection and modulation of internally generated objects (see “Internal dimension of listening effort”). This directional taxonomy of attention provides the most natural framework for our conceptual vectorial model of effortful listening in which the associated listening demands have different directions. We will complement this vectorial model with a computational representation of “attentional effort” (see Sarter et al., 2006) to map a (constrained) listening effort response along the orthogonal axis of the internal and external demands, see “Effort as a response to attentional demands”.

### External dimension of listening effort

Let us take a closer look at listening situation (a) (speech in a noisy background). Here we have a setting in which many auditory streams are competing for further processing. Assume that we have one intended or goal stream (the presenter’s explanation) and several streams of distraction. Automatic, bottom-up, processes often represented in terms of principles of Gestalt theory organize sensory information and auditory objects, respectively, into coherent streams (“auditory scene analysis”) (Bregman, 1990; Wrigley & Brown, 2004) based on exogenous (physical) properties of the signal. Although these exogenously directed scene analysis mechanisms are important for speech perception in realistic environments, they are almost certainly automatic and thus unlikely to be effortful (e.g., see Bregman, 1990; Wrigley & Brown, 2004; Strauss et al., 2010; Haab, Trenado, Mariam, & Strauss, 2011; Schneider & Chein, 2003for a review of the classical *Shiffrin & Schneider (1973) theory*). Nevertheless, they figure heavily in some considerations of auditory attention (e.g., Shinn-Cunningham & Best, 2008) and must be considered here at least in passing.

Goal-directed endogenous (schema-based) top-down processing also affects this stream formation. We refer to this endogenous influence on auditory scene analysis as external attention—the endogenously motivated direction of attention to properties of the sensory stimuli. Of course, the direction of external attention may also be affected by the output of processes that involve directing internal attention as well, but as long as the targets of this attentional direction ultimately consist of representations of external phenomena, we consider this to be external attention (Wrigley & Brown, 2004; Strauss et al., 2010), see below. Neurophysiologically, external attention modulates the flow of information along the auditory pathway by means of both corticocortical and corticofugal projections (see Trenado, Haab, & Strauss, 2009; Strauss et al., 2010 and references therein). In addition to such modality specific modulation, external attention also binds simultaneously presented information into multisensory objects and a coherent representation of the multisensory environment at supramodal processing stages, see Engel et al. (2001), Fougnie (2008), Chun et al. (2011). However, accounting for multisensory integration would overburden these initial steps to develop a taxonomic model of listening effort, and therefore we focus exclusively on the auditory modality in our further discussion.

In the probabilistic auditory stream selection model in Strauss et al. (2010), auditory streams are associated with exogeneous (physical properties of the sound stimulus) and endogenous (schema-based information) weights which determine their probability of being selected. Exogenous weights are influenced by external factors, e.g., stimulus intensity, and thus are presumably not associated with effort while endogenous weights are influenced by internal factors including especially the distribution of selective (external) attention (which is associated with effort). In terms of the *biased competition theory* (Desimone & Duncan, 1995) developed in visual perception, these exogenous and endogenous weights determine the bias of a given auditory stream. Using this framework, we call the goal or intended stream (the presenter’s speech) the *matched stream*. If the bias given to the matched stream through the combination of endogenous and exogenous weights is large as compared to the bias for each of the distractor streams, there is a small external demand of effort—successful segregation of this speech from the surrounding distractors can be accomplished relatively easily.

In the case of our listening situation (a) background noise, exogenous weights given to the matched stream would be high if the presentation is clearly separated from the other streams in terms of stimulus and environmental properties, i.e., spatial location, fundamental frequency, timbre or rate, etc. Endogenous weights, on the other hand, might be high because the contents of the matched stream are of special significance to the listener. If instead the exogeneous weights of the matched stream were in same the range as those of the other streams (i.e., because of similarities in physical properties), bias toward the matched stream would be low, its probability of being processed would, all else being equal, be quite similar to that of the other streams, and performance would be quite low. In this case, the listener might choose to “amplify” the bias by increasing endogenous weight given to the matched stream using an effortful increase of external attention in space and time, e.g., by exerting a more intense goal-directed spatial cuing—resulting in a larger expenditure of external attentional effort to achieve the same level of message understanding as in the case in which exogenous weights were higher. Thus, the endogenous application of external attention (i.e., paying attention to a particular location in space, a particular pitch range, etc.) can serve to improve selection of a matched stream, improving performance over the case of listening in the same conditions but with less effort being expended. In both cases, however, it is important to remember that these endogenous and exogenous weights relate to the biasing of external attention (i.e., object formation/stream selection) only.

### Internal dimension of listening effort

The external demand and effort described above are related to the selection and modulation of perceptual objects/streams coming through the auditory system. Thus, they play a much more minor role in listening situation (b) speech that is intrinsically difficult to understand. Here the matched stream is clearly audible and there are no distracting streams at all. Thus, there is little need to devote external attention to the listening task. However, this listening situation requires a large amount of internal or central (Fougnie 2008; Chun et al. 2011) attention. This internal attention operates over representations in working memory, long-term memory, task rules, decisions, and responses. In other words, it constrains operations that are focused on these type of cognitive or internal processes.

Capacity limitations of course also hold for internal attention. There is evidence that the capacities related to internal and external attention might be independent, see Potter, Chun, Banks, and Muckenhoupt (1998), Arnell and Jolicoeur (1999) as reviewed by Chun et al. (2011) though this is not a necessary condition for our conceptual model in “Conceptual vectorial model” as it maps conjoint capacity models (e.g., see Kiyonaga & Egner, 2013) too. In listening situation (b) the reconstruction of meaningful sentence or the semantic induction of unknown words certainly implies an increased working memory demand. In more general terms, linguistic–conceptual challenges result in an increased central or internal demand and thus require an increased internal effort to cope with the listening situation. It goes without saying that internal attention is of course also rather a catch-all term. It is still unclear whether there is a core central mechanism that governs all executive functions, or whether there are specific mechanisms for different domains of central attention, see, e.g., Wojciulik, Kanwisher, and Driver (1998), Badre and Wagner (2004), Raz and Buhle (2006), January, Trueswell, and Thompson-Schill (2009).

It is also worthwhile to emphasize that the internal and external dimensions may interact in sensory processing, e.g., for providing the top-down modulation that is required for biasing the selection of information in competition for processing resources (Chun et al. 2011). For example, a listener might begin to direct more external attention to focus on the speech of one talker in a crowd after determining that the application of internal attention to that person’s speech was still insufficient to successfully understand what they were saying. Conversely, a listener might choose to withdraw attention from the voice of a talker that is difficult to distinguish from the surrounding hubbub after realizing that what they are saying is quite easily predicted from context and prior knowledge. In addition to such reciprocal trade-offs, at a minimum, working memory has to be considered as a segregated internal process (Rowe, Toni, Josephs, Frackowiak, & Passingham, 2000; Postle, 2006) subserving both perceptual and cognitive processes (Fougnie, 2008; Chun et al., 2011), see also Joseph et al. (2016) for a recent review of resource allocation models of auditory working memory. However, even though there may be considerable functional overlap among the mechanisms that support the allocation of internal and external attention, there is converging evidence that external and internal (or perceptual and central) demands represent different dimensions of attentional processing and, therefore, as we argue in the next section, also of listening effort.

### Effort as a response to attentional demands

Sarter et al. (2006) stated “increases in attentional effort are defined as the motivated activation of attentional systems in response to detrimental challenges on attentional performance such as the presentation of distractors, prolonged time-on-task, changing target stimulus characteristics and stimulus presentation parameters, circadian phase shifts, stress or sickness” (p. 145). Thus the attentional response to changing external and internal task demands is not invariant over parameters such as vigilance and arousal level, motivation, or individual differences in actual available capacity, especially in multimodal settings. In other words, even for the very same external and internal task demands, the response in terms of exerted listening effort might be different as other conditions vary. Thus the effort exerted in the very same poster session cases mentioned before could easily vary depending on external context. For example, exerted effort may be very low if you are suffering from heavy jetlag or very high if the presented material is of major importance to your subsequent talk. This takes us to the problem of motivation, arousal, and fatigue which are intimately related but not identical to effort.

Attentional effort is clearly related to motivation (cf. Sarter et al., 2006), but there are important distinctions. For example, typically people seem to be strongly motivated to avoid effort (i.e., they try to find the easier way to solve a problem) and in fact as experimental scientists we necessarily assume that participants in our experiments never expend more effort than they have to solve a particular task (cf. Kahneman, 1973). On the other hand, there are certainly familiar cases in which people deliberately seek out effortful tasks, such as when one might opt to solve the more difficult Sudoku puzzle over the easy one precisely because it is more effortful and hence more engaging (cf. the concept of “flow” Nakamura & Csikszentmihalyi, 2014) and under many circumstance the exertion of effort has strong relevance for survival (cf. Kurzban, 2016) even though it is fatiguing. Thus, although motivation and fatigue are increasingly being seen as a crucial factor in understanding how listening effort affects human behavior (Hornsby et al., 2016; Mackersie & Calderon-Moultrie, 2016), they are not identical to effort.

Similarly, it has been clear since at least (Kahneman 1973) that the difficulty of a given task can modulate the availability of attentional capacity (modeled as arousal by Kahneman, 1973) such that it is possible to allocate more attention to difficult tasks than to easy ones, and yet it is impossible to allocate more than the necessary amount of attention to a given task. For example, when given a set of simple math problems that one can solve perfectly with little effort, it is impossible to “work harder” than the minimum required to accomplish the task perfectly; nevertheless, increasing the difficulty of the problems permits an increased allocation of attentional effort to the task. However, despite this, task difficulty cannot account for all aspects of listening effort because there exist many kinds of tasks (“data-limited” in the sense of Norman & Bobrow, 1975) in which the difficulty of the task can be shown to affect performance but increasing effort is not effective and may not even be possible. Consequently, apart from the decomposition of attention into internal and external components, a conceptual model of listening effort must also respect the dynamic and state-dependent relationship between demanded effort and exerted effort, a relationship that ultimately depends on a variety of factors including, but not limited to, motivation, arousal (e.g., due to task demands) and fatigue (Richter 2016; Hornsby et al. 2016).

In summary, effort derives from the need for, or act of, allocating attention but, as we will discuss in another section, the precise details of how attentional demand relates to the sensation of effort may be relatively simple (effort reflects task-related consumption of a limited cognitive resource such as working memory capacity) to relatively complex (e.g., incentive- and opportunity cost-based models). In every case, however, it is necessary to be able to represent the relationship between the effort demanded by a particular task in a particular context, and the effort that a listener ultimately exerts. Here, we will represent this relationship in terms of a simplified demanded-exerted listening effort response as described in the next section. The relation of this demanded-exerted listening effort response to more general models of cognitive effort is then discussed in “Relation to other selected models and concepts”.

## Conceptual vectorial model

In this section, we establish a conceptual model to study effortful listening based on the aforementioned concepts. This model is embedded in a simple mathematical framework which enables a more quantitative approach to future research. The goal is to enable the computational simulation of effortful listening by modulating representations of internal and external demands by means of a parameterized resource-limiting function. This limiting function represents a compilation of various findings in the relevant literature into a single, consistent quantitative model of resource limitations. The choice of this particular, single function is motivated by simplicity. Future iterations of the model may incorporate different limiting functions, or even multiple functions representing different factors that affect the availability of processing capacity if, for example, such changes were warranted based on the results of future experiments and the demand of specific modeling tasks. Even though this initial model is relatively simple, our expectation is that quantitative observations of the behavior of this model might provide postdictions of experimental results from research on effortful listening, and/or may motivate the design of new experiments to generate such results in the future. Due its parameterized nature, such a quantitative model might be used in a variety of ways, for example to develop explanations of individual differences in effortful listening, or to generate testable predictions regarding the expected behavior of participants based on different types of resource-limiting models, or to explore the implications of various formulations of motivational aspects of listening effort (see “Observations from the taxonomic model” for details).

As we have discussed, external and internal demands have different targets and perhaps also different capacities. Thus we model them as independent dimensions of the overall demand of listening effort. One way of modeling independence mathematically is by the concept of orthogonality. Thus we assume that the external and the internal demand are mutually orthogonal, quantifiable by the nonnegative real numbers *d*_*e*_ and *d*_*i*_, respectively. Using this notation, the overall demand can be represented by the vector $\vec d\in {\mathbb {R}}_{+}^{2}$ with $\vec d=(d_{e},d_{i})^{T}$ (${\mathbb {R}}_{+}$ denotes the space of nonnegative real numbers). The *demanded listening effort* *ξ*_dem_ to solve an auditory task can thus be modeled as a (scalar) function $ \xi _{\text {dem}} : {\mathbb {R}}_{+}^{2} \mapsto {\mathbb {R}}_{+}$ of the overall demand $\vec d$ such that

1 $$ \xi_{\text{dem}}(\vec d)=\|\vec d\|_{2}=\sqrt{{d_{e}^{2}}+{d_{i}^{2}}}. $$

**Fig. 1:**
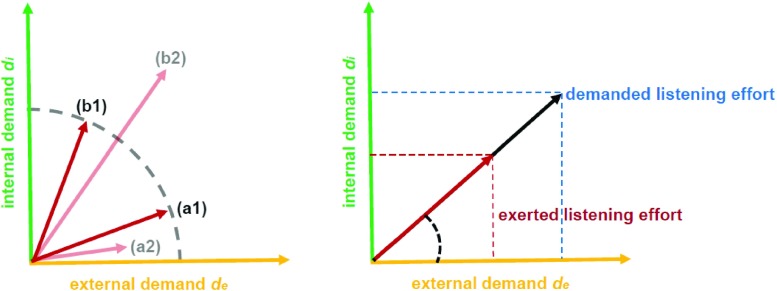
*Left*: listening effort as result of the internal and external demand (shown along the demand direction). Cases (a1) and (b1) map the situation (**a**) and (**b**) in our poster session example in “Two distinct dimensions of attention in effortful listening” for a constant overall effort but different internal and external demands. For the cases (a2) and (b2) the overall effort is different as well as the internal and external demands. *Right*: the exerted listening effort response (shown for illustration purposes also along the demand direction) follows also the demand $\vec d$ but is smaller than the demanded listening effort because of a resource-limiting function Υ (see text)

In other words, the demanded listening effort is just the Euclidean norm (or vector length) of the overall (vectorial) demand $\vec d$, see Fig. 1.

### Exerted Listening Effort

It is obvious that this demanded listening effort can differ from the *exerted listening effort* which has to respect capacity limitations, vigilance and arousal levels, as well as the motivation to exert effort, see Fig. 1 and also “Effort as a response to attentional demands”. As a first intuitive but computationally rigid step, we model these parameters by a bounded decaying function ${\Upsilon }_{p_{1},\ldots ,p_{n}}: {\mathbb {R}}_{+} \mapsto {\mathbb {R}}_{+}$ which we call a *resource-limiting function*. Here *p*_1_,…,*p*_*n*_ represents an abstract set of *n* parameters which makes it possible to model, e.g., different motivation or arousal levels. The exerted effort to such a resource-limiting function becomes

2 $$ {\xi_{\text{exe}}^{\Upsilon} (\vec d)= {\Upsilon}_{p_{1},\ldots,p_{n}} \left( \xi_{\text{dem}}(\vec d) \right) = {\Upsilon}_{p_{1},\ldots,p_{n}} \left( \sqrt{{d_{e}^{2}}+{d_{i}^{2}}} \right).} $$

**Fig. 2:**
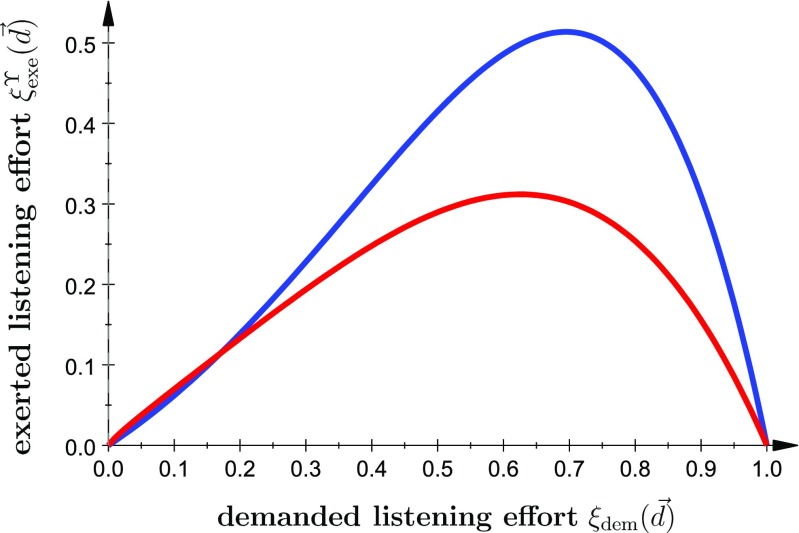
A possible example for the resource-limiting function ${\Upsilon }_{p_{1},\ldots ,p_{n}}(\cdot )$ which models the demanded-exerted effort response. Here we used ${\Upsilon }_{p_{1},p_{2}}(x)=\frac {{p_{1}}^{p_{2}}\, x^{{p_{2}} - 1}\, \mathrm {e}^{p_{1}\, \left (x - 1\right )}}{\Gamma \left (1 - x\right )}$ with Γ(⋅) being the Γ function as model (*x* ∈ [0,1]). For the blue curve we used *p*_1_ = *p*_2_ = 2 and for the red curve *p*_1_ = 1.4 and *p*_2_ = 1.85. Thus the parameters *p*_1_ and *p*_2_ control the steepness and intensity of the demanded-exerted effort response in this example. One could consider the *blue curve* as model for a high motivation whereas the *red curve* resembles rather a low motivation; with a less intense response (modeled by p1) and an earlier “quitting point” in the sense described before

A possible example for such a simple function with two parameters, i.e., *n* = 2, is shown in Fig. 2. This function responds almost linearly for small demands and decays quickly after a certain threshold of the demanded effort is reached. This morphology resembles earlier experimental results, e.g., the situation in which one just quits and elects not to solve a listening task (i.e., stops paying attention) because of too large internal/and or external demands, see Damian, Corona-Strauss, Hannemann, and Strauss (2015), Bernarding, Strauss, Hannemann, Seidler, and Corona-Strauss (2015), Pichora-Fuller et al. (2016), Richter (2016), and Bernarding, Corona-Strauss, Hannemann, and Strauss (2016). It also reflects the capacity supplied – capacity demanded curves according to (Kahneman 1973). Besides being just bounded because of capacity limits, this resource-limiting function Υ might also model, e.g., the dependence of effort on arousal level as given by the *Yerkes–Dodson law* (Yerkes and Dodson 1908; Kahneman 1973).

### Nonstationary Regime

It is important to emphasize that we may either assume a stationary task (i.e., one that does not change over time) or, more realistically, we may consider that *ξ*_exe_ is just an instantaneous representation of the time-varying resource-limiting function of a listener engaged in an ongoing listening task. In a nonstationary regime, in which the demand $\vec d$ depends on time $t\in {\mathbb {R}}$ (changing task demands over an experiment), a simple model for a time-dependent resource-limiting function yields

3 $$\begin{array}{@{}rcl@{}} \xi_{\text{exe}}^{\Upsilon} (\vec d(t))&=& {\Upsilon}_{p_{1}(t),\ldots,p_{n}(t)} \left( \xi_{\text{dem}}(\vec d(t))\right)\\ &=& {\Upsilon}_{p_{1}(t),\ldots,p_{n}(t)} \left( \sqrt{d_{e}(t)^{2}+d_{i}(t)^{2}}\right). \end{array} $$

**Fig. 3:**
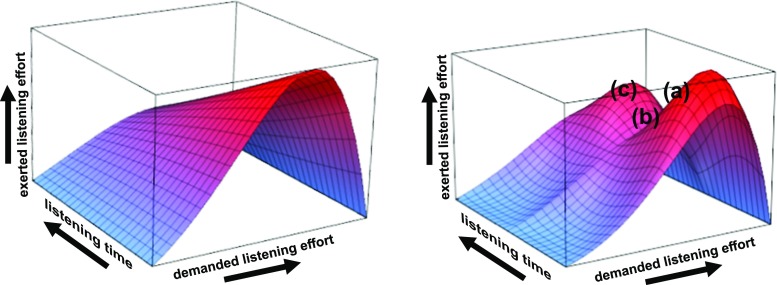
Two examples for a time-dependent resource-limiting function. Here we used the same model as in Fig. 2 but with *p*_1_(*t*) = 2 − *t* and *p*_2_(*t*) = 2 at the left and $p_{1}(t)=\mathrm {e}^{- 2\pi \, t} + 2$ and $p_{2}(t)=\left |\sin \!\left (2\pi \, t\right )\right | + 2$ at right hand (*t* ∈ [0,1]). Whereas the left surface represents a simplified decrease in arousal over the listening time, the right surface maps, e.g., a more complex modulation of exerted attentional effort based on a combination of the limiting/motivational factors over time. For instance: While there remains the same overall decline in exerted listening effort over time, at point (**a**) there is a momentary decrease in interest/motivation to listen, perhaps due to distraction; at point (**b**), a salient keyword (re)captures interest which results in (**c**) a momentary increase in the motivation to exert listening effort that is nevertheless still muted somewhat by the time-dependent decrease in arousal. Note that, although the exogenous capture of attention by the keyword may itself be effortless (see Westbrook and Braver, 2015), recognition of a relevant word and the resulting change in motivation may nevertheless induce a change in the subsequent allocation of attentional effort

It is important to note that the exerted effort depends not only on time but also on the parameters of the resource-limiting function ${\Upsilon }_{p_{1}(t),\ldots ,p_{n}(t)}(\cdot )$ itself. This reflects changing amounts of the available capacity and/or the influence of time-varying properties of vigilance, arousal and/or motivational levels during a listening task. This is conceptually shown in Fig. 3.

### Distinct Capacities

We can also model the resource-limiting function Υ independently along the external and internal dimension, e.g., to map the independent capacities. In this case, it becomes a vector valued, yielding the scalar-valued exerted listening effort by

4 $$ {\xi_{\text{exe}}^{\vec {\Upsilon}} (\vec d(t)) = \sqrt{{\Upsilon}^{e}_{p_{1}(t),\ldots,p_{n}(t)}(d_{e}(t))^{2}+{\Upsilon}^{i}_{q_{1}(t),\ldots,q_{m}(t)}(d_{i}(t))^{2}},} $$

with ${\Upsilon }^{e}_{p_{1}(t),\ldots ,p_{n}(t)}(\cdot )$ and ${\Upsilon }^{i}_{q_{1}(t),\ldots ,q_{m}(t)}(\cdot )$ being the internal/ external vector components of a (parameterized) vector-valued resource-limiting function $\vec {\Upsilon }_{p_{1}(t),\ldots ,p_{n}(t),q_{1}(t),\ldots ,q_{m}(t)}: {\mathbb {R}}_{+}^{2} \mapsto {\mathbb {R}}_{+}^{2}$. Note that the number *n* of the *p*-parameters might differ from the number *m* of *q*-parameters as different functions might be used to model internal and external limits. Thus, the complete model maps a nonstationary demanded-exerted effort response, decomposed along the orthogonal internal and external dimension by means of a vector-valued resource-limiting function. Note that we can also work with the individual components ${\Upsilon }^{e}_{p_{1}(t),\ldots ,p_{n}(t)}(d_{e}(t))$ and ${\Upsilon }^{i}_{q_{1}(t),\ldots ,q_{m}(t)}(d_{i}(t))$ if a vectorial mapping of the exerted listening effort is preferred.

Given that the various iterations of the model discussed here vary considerably in terms of complexity, determining which of the above models should be applied in a given case will of course depend on the scale of detail needed for a particular application. Note also that the use of rigid functions is merely a preliminary, intuitive step to model resource limits. It goes without saying that more advanced and flexible dynamic models are possible here, see “Resource-Limiting Models” in “Observations from the taxonomic model” which map active listening situations more accurately and adaptively. However, even in this simplified representation, there are several observations that can be derived by observing properties of the taxonomic model which we summarize in the next section.

## Observations from the taxonomic model

The taxonomic model of attention in effortful listening described here is intended to provide a useful starting point from which to further investigate the many possibly distinct phenomena currently grouped under the catch-all term ’listening effort’ within a more differentiated, cognitive neuropsychological framework. It remains to be seen, of course, to what degree a model can be considered accurate when it makes use of abstract constructs such as attention and effort. However, following the arguments of Chun et al. (2011), we believe that the major contribution of such a taxonomic model is not whether or not it is explicitly correct in every detail, but rather that it serves to emphasize a distinction between two types of processes which appear, based on our current knowledge, to be plausibly segregatable in effortful listening. We employed the concept of orthogonality in our conceptual vectorial model to map these different processes in terms of different dimension of an active listening task.

In addition to thereby instantiating a taxonomic model of attention (Chun et al. 2011), we include the abstract construct of a resource-limiting function that allows us to represent the dependence on resource limitations that represent effects of fatigue, arousal levels (Kahneman 1973) and/or motivation in the sense of Sarter et al. (2006). As these functions can also be nonstationary, the possible dynamic modulation of effortful listening by motivation, arousal, and vigilance can be represented, too. Due its vectorial nature, our model allows for an easy graphical interpretation which might support its applicability to a wide range of problems in auditory processing and perception. For example, the model might be applicable to accounting for patterns of existing results, e.g., the divergence between measures of subjective effort and objective scaling for too small and/or too large external demands (e.g., see Damian et al., 2015; Bernarding et al., 2015, see also discussions by McGarrigle et al., 2014; Pichora-Fuller et al., 2016), and see additionally “Relation to other selected models and concepts”). However, here we rather want to focus on some observations from the model which might be useful to derive predictions that can be confirmed or disconfirmed experimentally, thus stimulating future research.

### Experimental Design

The model suggests the possibility of designing experiments that specifically address the internal and external dimension of effortful listening. We have based this aspect of the model on extrapolation and conjectures derived from the results of existing research on attention and effort, but the need for this distinction in the realm of listening effort remains to be demonstrated experimentally. The presented taxonomic model might therefore guide further electromagnetic or metabolic neuroimaging experiments designed to confirm or disconfirm the suggested orthogonality of the dimensions of the demand, perhaps by simulating specific properties of the two distinct poster session problems stated before, either in an auditory free field or in a reduced version via headphones. Such experimental designs require manipulations of the signal and the task at the same time and are most productively explored using an interdisciplinary approach combining hearing and cognitive research, an approach that is already common in research on the effects of age on speech perception as exemplified by e.g., the research programs of Humes and colleagues (Humes 2013), Pichora-Fuller and colleagues (Schneider et al. 1995), Schneider and colleagues (e.g., Schneider, Avivi-Reich, & Daneman, 2016) and Wingfield and colleagues (e.g., Wingfield & Tun, 2001, 2007), among others.

### Iso-Effort Lines

One implication of the model is that there exist “iso-effort” lines reflecting equivalencies of the exerted listening effort $\xi _{\text {exe}}(\vec d)$, e.g., see the quarter circle in Fig. 1, left hand, case (a1) and (b1). Here the exerted listening effort is the same in the two conditions (a1) and (b1), but the contribution from internal and external attentional effort is different for each one. Even though these iso-effort lines are purely theoretical constructs, they might help to explain diverging results for estimates of exerted listening effort that arise when using different methods to assess effort.

For example, discrepancies may often be observed when comparing measures of subjectively experienced effort (i.e., self-report) with different types of objective measures (i.e., psychophysiological measures, or measures of dual-task performance) (cf. McGarrigle et al., 2014). It might be that some techniques tend to reflect overall exerted effort, i.e., the magnitude of the exerted effort vector irrespective of the relative contributions of each of the contributing dimensions, whereas other methods are more sensitive to one or the other of the dimensions in the provided taxonomy. Similarly, it seems quite plausible that individuals may vary in terms of their subjective response to demands on internal vs. external attention, such that, for a particular individual, two listening conditions that are equivalent in terms of overall demand (i.e., they both lie along the same iso-effort line) are nevertheless perceived (and subjectively rated) as differently effortful because one involves much higher demand on one dimension than does the other. Research in this direction might begin with attempts to equate the subjective effort of different tasks designed to differ mainly in their relative demand on either internal or external attention.

### Differentiated Measurements

It seems likely that some physiological or behavioral measurement techniques may be more sensitive to the contributions of one demand dimension over another. In this case, such dimension-dependent techniques might be applied to analyze effortful listening in a more differentiated manner than is currently pursued, permitting researchers to employ demand-specific methods to focus investigations on the contribution of either internal or external attentional effort as needed. Such targeted methods will depend first on determining the sensitivity of particular techniques to demands on the external or internal dimension, but once this has been done for a range of methods it will become possible to select measurement strategies with a much greater degree of precision. This knowledge would be of particular importance when attempting to objectively assess listening effort for specific purposes for which only one of the two dimensions might be relevant, for example in order to optimize hearing systems (Downs 1982; Sarampalis et al. 2009; Strauss et al. 2010; Bernarding et al. 2013; Pichora-Fuller et al. 2016; Bernarding et al. 2017) or auditory human–machine interfaces (Damian et al. 2015).

### Individual Differences in Resource Limitations

The concept of a resource-limiting term which maps how exerted effort follows the demanded effort is rather flexible. This demanded-exerted effort response, especially in its nonstationary variant, might be affected by a variety of individual and contextual characteristics such as motivation, attentional control, and arousal. The construct of a resource-limiting function might also provide insight into prior results.

For example, such functions may be fit to previously established dynamic effort assessments to better understand how the availability of attentional resources varies over time and across individuals. Moreover, when using a vector-valued variant, resource-limiting functions allow modeling of internal (central) and external (perceptual) limits separately, irrespective of whether these limits reflect different capacities for separate pools of resources, or simply distinct allocation schemes for a shared resource such as *supramodal attention* proposed by Kiyonaga and Egner (2013). Such a separation could facilitate modeling studies designed to tease apart the differential contributions of perceptual and central factors to listening effort, for example to attempt to distinguish between the relative contributions of age-related changes in audition vs. cognition to age-related increases in listening effort, see Wingfield et al. (2005).

Extending this to more prospective studies, it might be interesting to explore the degree to which the effort associated with more perceptual/sensory integration (i.e., external attention applied to object formation and selection) might be related to effort associated with other sorts of integration, e.g., the integration of new sensory representations into existing (longer-term) knowledge structures, see Hannon and Daneman (2014).

### Implications for the Study of Listening Fatigue

Sustained listening effort in hearing impaired persons and its relation to fatigue concepts is an active area of research, see Hornsby et al. (2016). However, as Hornsby et al. (2016) point out, the mechanisms which cause hearing loss-related fatigue as well as the efficacy of audiologic interventions for reducing this type of fatigue are unclear. The model presented here, with its mutually orthogonal internal and external components, suggests that external (perceptual) and internal (central) components of demand might contribute differently to fatigue or, more precisely, to the quantification of fatigue-related factors by different assessment techniques. Thus the assessment of the demanded-exerted effort response over time might also help us to better understand the relation between listening fatigue and listening effort.

### Resource-Limiting Models

In the proposed model, we used rather rigid functions to model resource limitations as an intuitive starting point. It seems quite likely that more advanced resource-limiting models may be of value, for example to quantitatively map in detail the interaction of intrinsic capacity limits, motivational aspects, nonstationary vigilance and arousal levels (related to both task-specific demand and to individual differences) or by making use of probabilistic concepts to model these variables. More macroscopically, individual differences might be investigated in terms of differences in resource-limiting terms, a topic for further interdisciplinary research at the interface of neuropsychology and computational neuroscience. Computational research might also include a further decomposition of the proposed external and internal components of such functions after a more differentiated analysis of these dimensions is available.

In general, research in speech perception is marked by a paucity of models that incorporate resource limitations as an intrinsic property of the system (Heald & Nusbaum, 2014), so by providing a straightforward model for quantifying the contributions of different sub-components to the overall demand of a listening task, the present taxonomic model may serve as a starting point for developing more quantifiable models of the role of resource limitations in speech perception in a more general sense. In particular, when using a functional programming representation, an active listening task such as speech perception can be modeled as a constrained dual optimization problem (maximizing speech perception outcomes while minimizing the exerted effort) with motivation-driven objective functionals that depend on the defined vectorial demand and having the resource limitations as constraints. While the details of such a characterization must be left for subsequent work, even the more conceptual model presented here can be related to a variety of major theories addressing the intersection between limited resource capacity, selective attention, speech perception, and listening effort, as discussed in the following section.

## Relation to other selected models and concepts

The proposed model, in which externally and internally directed attention are governed by essentially orthogonal systems, can also be related to a number of other attentional models, although in some cases the similarity may turn out to be mostly superficial. Here we address six prominent theories (or classes of theories) that may be related to the model presented here.

### Perceptual Load Theory

Lavie and colleagues distinguish between the effects of perceptual and cognitive load on interference from distractors (Lavie et al. 2004; Lavie 2000), arguing that conditions of high perceptual load (i.e., a target presented amongst many irrelevant distractors) lead to efficient exclusion of distractors from subsequent processing, while conditions of high cognitive load (i.e., increased demand on working memory) result in increased distraction. However, it must be noted that, in Lavie’s work, the effects of both perceptual and cognitive load are discussed in terms of their effects on the success of selecting external (sensory) objects. That is, we would say that they both affect the allocation of external attention, albeit via different mechanisms (perceptual vs. working memory load). As discussed above, the conceptual model proposed in the present article is entirely compatible with the possibility that working memory serves as a limiting factor on the allocation of not only internal but also external (i.e., sensory object-directed) attention as proposed by Lavie and colleagues. Furthermore, the present model focuses on the active allocation of attentional resources (attentional effort in the sense of Sarter et al., 2006), and is not intended to address the automatic reduction in distractor interference described by Lavie and colleagues as a consequence of increased perceptual load (which may (Fairnie et al. 2016; Francis 2010) or may not (Murphy, Fraenkel, & Dalton, 2013) obtain in the auditory domain). Thus, there is no inherent disagreement (but also no necessary agreement) between the model of attentional effort proposed here and the differential effects of perceptual and cognitive load on attentional selection of sensory objects (and, thus, external attentional effort) as described by Lavie and colleagues.

### Active Speech Perception Models

Similarly, Nusbaum and colleagues (see Francis & Nusbaum, 2009; Heald & Nusbaum, 2014) have argued that listeners’ ability to cope with the effects of reduced perceptual acuity (i.e., when listening to masked or distorted speech) depends on the availability of working memory resources because poor sensory representations of speech tokens (e.g., lexical items) activate a larger number of potentially valid representations (interpretations) that must be maintained in working memory until a correct interpretation is determined. Although Nusbaum and colleagues do not specifically address the determination of effort within the context of their model, their model does depend crucially on the commitment of a limited-capacity resource (working memory or attention), and as we have discussed, listening effort may be most succinctly linked to the goal-directed allocation of such resources. Once again, the taxonomic model proposed here is not incompatible with the idea that limits on working memory or some other cognitive capacity constrain the ability to evaluate multiple competing interpretations of an ambiguous sensory stimulus.

In this case, the task described by Nusbaum and colleagues as selecting between multiple internal representations of a single stimulus could be characterized as an exercise in the allocation of internal attention. Thus, while the effects of both perceptual and cognitive load described by Lavie and colleagues would seem to both pertain primarily to the allocation of external attention (and thus primarily affect external attentional effort), in the case of the model proposed by Nusbaum and colleagues, any increase in listening effort associated with degraded sensory input (requiring the generation of a greater number of potential interpretations to be maintained in working memory) would seem to be most plausibly associated with the allocation of internal attention. One benefit of the taxonomic model presented here may be that, although both sorts of limited-resource effects can be accommodated within the parameters of the model, by distinguishing between the allocation of attention to external vs. internal objects, the taxonomic model may permit a more refined range of predictions regarding the specific neural systems underlying these potentially distinct aspects of listening effort.

### The Ease of Language Understanding (ELU) Model

The ELU (e.g., Edwards, 2016; Rönnberg, Rudner, Foo, & Lunner, 2008; Rönnberg et al., 2013) was initially developed in large part to account for the way in which distortion of the peripheral auditory signal, especially through the presence of noise and/or hearing loss, seems to contribute to increased cognitive processing demands for understanding speech. For our purposes here, the most significant property of the ELU is its architecture, especially that proposed by Edwards (2016).

Initial conceptualizations of this model (e.g., Rönnberg et al., 2008) were purely feed-forward and required the speech processing mechanism to explicitly switch between processing modes when auditory representations of the incoming signal could not be matched to long-term representations of speech. Specifically, processing was presumed to shift from an automatic, effortless mode to an effortful mechanism that incurs demand on working memory capacity. Subsequent development of the model introduced a feedback mechanism (Rönnberg et al. 2013) and the most recent model (Edwards 2016) incorporates an additional, earlier, effort-demanding module for “auditory scene analysis and attention”, plus the inclusion of a feedback pathway providing the potential for influence on this earlier processing system from the later-occurring, effort-demanding explicit processing module. This version of the ELU thus explicitly incorporates distinct modules for two kinds of effortful processing that map relatively well onto the concepts of external and internal attention in our model, and also permits for the interaction between internal and external attentional processing as described in “Internal dimension of listening effort”.

Like the present model, the hybrid ELU model proposed by Edwards (2016) also remains uncommitted with respect to whether the allocation of capacity to these two types of processes draws on a single source or distinct ones. Thus, the model we propose here is quite compatible with the most recent iteration of the ELU, but also goes further by making explicit the relationship between the two types of attentional effort (external and internal) and the global sense of effortful listening. It is also more directly compatible with current neurodynamic models of top-down sensory processing.

### Models Integrating Selective Attention and Working Memory

Recent characterizations of attention have begun to consider the possibility that internal and external attention, may both simply reflect the application of the same fundamental resource to mental representations from different domains, one within the observer and one without. For example, Kiyonaga and Egner (2013) distinguish between “W[orking] M[emory] (internal attention) and perceptual selection (external attention)” (p. 3), but propose that the two systems depend on the allocation of a single pool of resources that they call “supramodal attention”. In terms of the model proposed here, such a single pool of resources can be easily modeled by Eqs. 2 and 3 in “Conceptual vectorial model” whereas Eq. (4) represents a condition in which resources are distinct.

Similarly, Nobre and colleagues (Nobre et al. 2004) distinguish between the “selection of items located ‘in the extrapersonal world’ and selection of internal “mental representations based upon experiences and expectations” (p. 363). Furthermore, they find a large (though not complete) overlap in brain regions activated during the direction of attention to both visually present (external) and no-longer present (internal) visual arrays, suggesting that the two types of attention share a common neural basis. We have made a strong case here that the direction of attention to these two distinct sorts of representations may have distinct influences on the sensation of listening effort itself. However, this is based primarily on the precedence set by existing treatments of listening effort and current models of attention. It remains to be seen whether tasks that differentially invoke demand on these two types of effort engage the same or distinct neural circuits.

As it stands, the model is nevertheless capable of representing the possible case in which (a) effort depends entirely on the amount of capacity (e.g., working memory) allocated to a task, and (b) external and internal attentional allocations are yoked in the sense of drawing on a single pool of resources. In this case, for any given pool of available capacity, the total capacity devoted to the task (and thus the effort exerted) would simply lie along a single *iso-effort line* as described above in “Observations from the taxonomic model”. Thus, the proposed taxonomic model can be used to generate hypotheses about effort under certain varying circumstances that may be used to test the applicability to the study of listening effort of theories such as that of Kiyonaga and Egner (2013) that attempt to base internal and external attention (and thus corresponding effort) on the allocation of a single pool of resources (e.g., working memory capacity).

### Models of Incentive and Opportunity Cost

Regarding neuroeconomic approaches to “effort” (see Westbrook & Braver, 2015), we recognize that there is a potentially important distinction to be made between characterizing attentional effort as “the motivated activation of top-down mechanisms to counter performance decline” (Sarter et al., 2006) and characterizing it as a response to an assessment of the opportunity cost related to such activation, i.e., “the output of mechanisms designed to measure the opportunity cost of engaging in the current mental task.” (Kurzban, 2010, cited in Kurzban et al., 2013, p. 665). However, resolution of this distinction is beyond the scope of the present work. As currently formulated, the model incorporates a single term for “motivation” which is consistent with the way in which motivation is discussed in existing models that have been applied to listening effort (e.g., Kahneman, 1973; Hornsby et al., 2016) and seems more compatible with the more strictly incentive-oriented general models such as that of Sarter et al. (2006).

However, we see no reason to rule out the possibility of expanding the motivation term to incorporate not just incentive but also some assessment of the value of alternatives to engaging in the task at hand, which would permit our model to reflect opportunity cost more accurately. In this case, the demanded-exerted effort response according to Eqs. 2–4 in “Conceptual vectorial model” can be interpreted as driven by opportunity costs in sense of Kurzban et al. (2013), Kurzban (2016). Here the response is just following the opportunity cost of engaging in the current listening task. This becomes particularly apparent when following the dynamics in Fig. 3 (right). However, we leave a detailed exploration of alternative interpretations to our “attentional effort” (Sarter et al. 2006) line of argumentation to future work.

### Neurodynamic Top-Down Processing Models

In recent years, respectable advances have been made in our understanding of neuronal mechanics underlying top-down processing in attention, working memory, and reward related behavior (e.g., see Engel et al., 2001; Grossberg, 2005; Sarter et al., 2006; Gilbert & Sigman, 2007; Ferenczi et al., 2016 for reviews). Some of these have a direct relevance for the presented taxonomy of effortful listening. In particular, Shinn-Cunningham (2008) translated ideas of the biased competition theory developed in visual perception (Desimone & Duncan, 1995) to the auditory domain to explain interactions between auditory selective attention and auditory object formation (see also work by Alain and colleagues, e.g., Alain & Arnott, 2000; Alain et al., 2013, supporting object-based attention in the auditory domain). Shinn-Cunningham (2008) suggested that the results of many studies on ’informational masking’ can be explained using this framework. Biased competition and other schemes of top-down processing, see Engel et al. (2001), Sarter et al. (2006) for a review, inherently distinguish between stimulus-driven (exogenous) bottom-up and goal-directed (endogenous) top-down processing. The model proposed here focuses on the second of these (cf. “External dimension of listening effort”), along with a third kind of mechanism consisting of cognitively demanding internal processes, e.g., “filling in” and “repair strategies” that have been identified as particularly relevant in research on auditory processing, especially of speech (Shinn-Cunningham & Best, 2008; Heald & Nusbaum, 2014). Thus, this model builds on the taxonomy proposed by Chun et al. (2011) to emphasize both similarities and distinctions between the goal-directed top-down processing considered in biased-competition theory, i.e., processing that targets the external dimension, and the goal-directed top-down processing that addresses internal representations, while distinguishing both from purely bottom-up processing that acts along the external dimension exclusively. In this sense, the present taxonomic model is compatible with the neurodynamical models of listening effort and selective auditory attention presented by Strauss et al. (2008), Strauss et al. (2010) which quantitatively map the balance between goal-directed top-down processing and pure bottom-up processing to large-scale neural correlates of corticothalamic feedback dynamics (Llinás, Ribary, Jeanmonod, Kronberg, & Mitra, 1999; Destexhe, 2000; Hillenbrand & van Hemmen, 2002; Robinson, Rennie, Rowe, O’Connor, & Gordon, 2005) and corticofugal modulation (Suga, Xiao, Ma, & Ji, 2002; Nuñez & Malmierca, 2007; Luo, Wang, Kashani, & Yan, 2008) along the auditory pathway.

While the present model is purely conceptual, in the sense that we have made no attempt to relate components of the model directly to neurological substrates, the potential for such a connection exists, and future iterations of the model could be adapted to better reflect developing insights into the function of specific neural circuitry. For example, Sarter et al. (2006) has suggested that top-down circuits mediate increases in attentional effort in vision in order to balance/attenuate the effects of detrimental challenges to visual performance (compare our arguments in “Effort as a response to attentional demands”), and has argued that these circuits are largely driven by increased activity in prefrontal cortical cholinergic inputs (Sarter & Bruno, 2000; Sarter et al., 2006). Control structures of this sort are currently represented in the model only in very general terms, but future research from both the modeling and the neurophysiological domains will make it possible to further refine this aspect of the model. Conversely, predictions based on the architecture and/or behavior of this model may also help to guide future neurophysiological research. For example, Westbrook and Braver (2016) discusses the role of dopaminergic neurotransmission in linking the operation of working memory to the sensation of internal effort (associated with the commitment of working memory capacity to goal-oriented processing, see also Kiyonaga and Egner, 2013), and to the evaluation of the effectiveness of that effort in the context of motivation. As currently formulated, the present model distinguishes between internal and external dimensions of effortful listening, but remains uncommitted as to the degree to which resource allocation and the evaluation of performance are distributed across these two dimensions. Future research might therefore aim to isolate neuronal correlates of the internal and external dimension of effortful listening across different spatiotemporal scales of neural processing, see also “Observations from the taxonomic model”.

## Conclusions

We have proposed a taxonomic model of attention in effortful listening. Due to the soaring interest in the catch all term “listening effort” in recent years, our hope is that the model will serve as starting point to facilitate the analysis of effortful listening within a more differentiated cognitive neuropsychological framework. The model maps explicitly the internal and external dimensions of effortful listening as well as the dynamic nature of resource limitations to reflect the influence of such variables as vigilance, arousal, and motivational levels. Due its vectorial nature, our model has a straightforward graphical interpretation and can thus be intuitively applied to a broad range of problems in auditory processing and perception. We provide some observations from the model which might serve to provide postdictions of existing results as well as to stimulate further research. As such the model complements and extends existing models and theories of attention and effort with particular relevance to understanding the phenomenon of effortful listening.
